# Coupled Contagion Dynamics of Fear and Disease: Mathematical and Computational Explorations

**DOI:** 10.1371/journal.pone.0003955

**Published:** 2008-12-16

**Authors:** Joshua M. Epstein, Jon Parker, Derek Cummings, Ross A. Hammond

**Affiliations:** 1 Center on Social and Economic Dynamics, The Brookings Institution, Washington D. C., United States of America; 2 Santa Fe Institute, Santa Fe, New Mexico, United States of America; 3 University of Pittsburgh Graduate School of Public Health, Pittsburgh, Pennsylvania, United States of America; 4 The Johns Hopkins University Bloomberg School of Public Health, Baltimore, Maryland, United States of America; Yale University, United States of America

## Abstract

**Background:**

In classical mathematical epidemiology, individuals do not adapt their contact behavior during epidemics. They do not endogenously engage, for example, in social distancing based on fear. Yet, adaptive behavior is well-documented in true epidemics. We explore the effect of including such behavior in models of epidemic dynamics.

**Methodology/Principal Findings:**

Using both nonlinear dynamical systems and agent-based computation, we model two interacting contagion processes: one of disease and one of fear of the disease. Individuals can “contract” fear through contact with individuals who are infected with the disease (the sick), infected with fear only (the scared), and infected with both fear and disease (the sick and scared). Scared individuals–whether sick or not–may remove themselves from circulation with some probability, which affects the contact dynamic, and thus the disease epidemic proper. If we allow individuals to recover from fear and return to circulation, the coupled dynamics become quite rich, and can include multiple waves of infection. We also study flight as a behavioral response.

**Conclusions/Significance:**

In a spatially extended setting, even relatively small levels of fear-inspired flight can have a dramatic impact on spatio-temporal epidemic dynamics. Self-isolation and spatial flight are only two of many possible actions that fear-infected individuals may take. Our main point is that behavioral adaptation of some sort must be considered.

## Introduction

*“The plague was nothing; fear of the plague was much more formidable.”*

Henri Poincare

### Motivation

In classical mathematical epidemiology–the tradition of ordinary differential equations with perfect mixing (mass action kinetics) beginning with the 1927 Kermack-McKendrick model–individuals do not adapt their contact behavior during epidemics [1]–[3]. They do not endogenously engage, for example, in social distancing (protective sequestration) based on disease prevalence. Rather, they simply continue mixing (often uniformly) as if no epidemic were under way. This may be a reasonable assumption for non-lethal infections such as the common cold, but for lethal diseases such as AIDS, it is known to fail; and for other lethal disease threats, like pandemic influenza or bioterrror smallpox, it seems likely to. People may be expected to adapt their contact patterns, and this will feed back to alter epidemic dynamics.

### Homo EconomSickus

Economists have begun to address this issue, introducing the notion of *prevalence elastic behavior* into epidemic models. For example, as AIDS prevalence grows in a community, people may reduce their number of sexual partners [4]. Predictably, *economic epidemiology*, as this subfield is called, posits optimizing behavior on the part of individuals. In effect, it models how canonically rational individuals would behave given some level of disease prevalence. They behave as *homo economicus* would behave given the associated health risks and costs of protection (e.g., vaccine-seeking). A term used for the resulting dynamics is *rational epidemics*. This literature includes elegant mathematical work, and captures—in the notion of prevalence elasticity—a clearly important phenomenon.

### Boundedly Rational Epidemics

However, prevalence is treated as a kind of exogenous signal (suspiciously like a perfectly competitive price) to which agents respond with some elasticity. They do not interact directly with one another to gain information on prevalence or in deciding how to behave. The approach, therefore, seems ill-suited to capture cases where *endogenous epidemics of fear inspire widespread adaptations unrelated to prevalence.* As an example, in 1996, millions of Indians fled Surat province to escape pneumonic plague. Yet, not a single case of pneumonic plague was actually confirmed. Prevalence of the disease itself, in other words, was zero.

We do not purport to define the term “fear.” Readers should feel free to interpret it as “concerned awareness,” for example. The point is that we are modeling a behavior-inducing transmissible signal distinct from the pathogen itself. For expository purposes, “fear” will do. For those interested in the substantial literature on emotional contagions generally, see [5].

The model developed here handles cases where the fear is contagious, even when the pathogen is not (e.g., anthrax). Indeed, it handles cases where the event in question is not a pathogen at all, such as a chemical or radiological event, or natural disaster, such as an earthquake or volcano.

A second problem with the literature is that, even models that *do* include prevalence-dependent behavior assume behavioral changes that are *depressive* in their effect on the epidemic - protective self-isolation (sequestration) being the most common. However, research on mass behavior during crises (and even epidemics specifically) records another behavioral response that is common - *flight*. Unlike protective sequestration, flight has the potential to *increase* long-range mixing across spatial regions, exacerbating epidemics. In the model introduced here, we expand the behavioral response repertoire of agents infected with fear to include both flight and protective self-isolation.

In summary, most infectious disease modeling ignores adaptive behavior. Models have begun to include prevalence elastic behavior [6]. It typically damps the epidemic. In Part I, we introduce a differential equation model where fear can spread independent of prevalence. Then spatial flight is added to the behavioral repertoire. This extension is implemented as an Agent-Based Model (ABM) in Part II. The full model shows how even a small amount of flight can amplify epidemic severity. To begin, we present the no-flight version.

## Analysis

### Part I: The Basic No Flight Model

For expository purpose, we imagine two *contagion* processes: one of disease proper, and one of *fear about* the disease. (Bear in mind that the model in fact does not require that the event sparking the fear epidemic be a disease, contagious or otherwise. It could be a radiological, or seismic event, for example.) Individuals contract disease only through contact with the disease-infected (the sick). However, individuals can contract fear through contact with the disease-infected (the sick), the fear-infected (the scared, or worried well), or those infected with both fear and disease (the sick and scared). Scared individuals—whether sick or not—may withdraw from circulation, and return to circulation having recovered from fear, all of which affects the course of the disease epidemic proper.

Agents can occupy one and only one of seven states at any time. The model's (seven dimensional) state space is shown below:

S: Susceptible to pathogen and fear
I*_F_*: Infected with fear only
I*_P_*: Infected with pathogen only
I*_PF_*: Infected with pathogen and fear
R*_F_*: Removed from circulation due to fear
R*_PF_*: Removed from circulation due to fear and infected with pathogen
R: Recovered from pathogen and immune to fear

Let 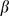 denote the per-contact disease transmission rate, and let α denote the per-contact fear transmission rate. If we now imagine a susceptible individual (i.e., neither sick nor scared) having contact with one who is both sick and scared, then the transmission rates of fear, infection, and various combinations are given in Table 1. For instance, the probability that the first individual (neither sick nor scared) contracts neither bug nor fear is 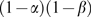, and so forth.

**Table 1:** Transmission Probabilities.

	Get scared	Not get scared
Get sick	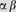	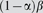
Not get sick	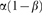	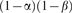

Finally we specify below the parameters controlling the rate at which individuals self-isolate due to fear and recover from fear and return to circulation:

λ*_F_*: Rate of removal to self-isolation of those infected with fear only
λ*_P_*: Rate of removal from infection with pathogen
λ*_PF_*: Rate of removal to self-isolation of those infected with fear and pathogen
H: Rate of recovery from fear and return to circulation

**Figure 1:**
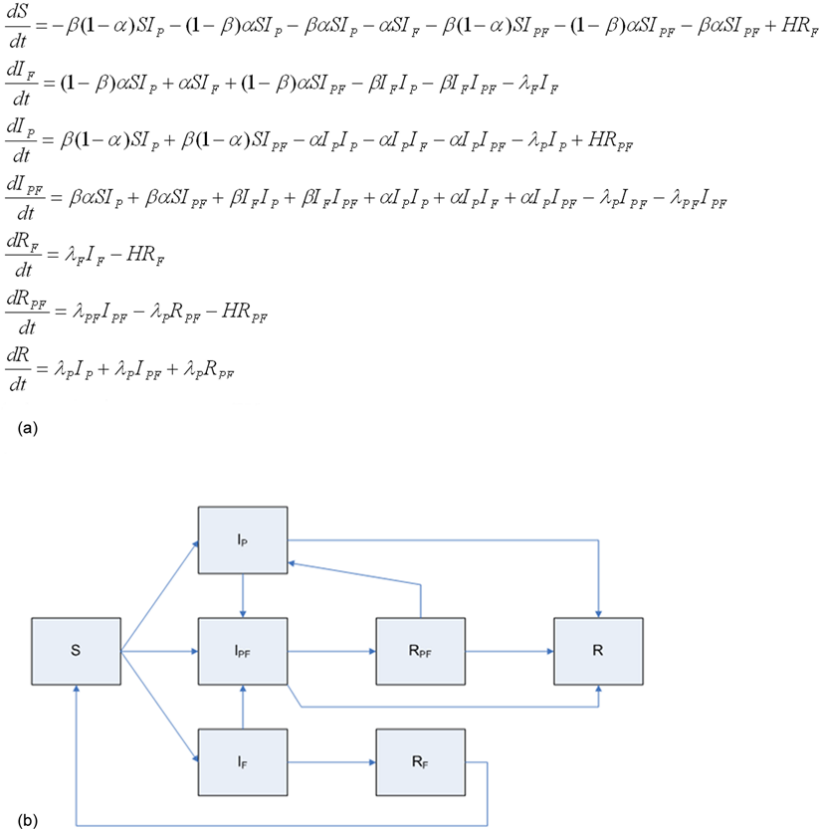
(A&B): Classical SIR differential equations formulation and flowchart.

With all of this in place, the model can be implemented as a classical well-mixed ordinary differential equation (ODE) system. The appropriate generalization of the standard Kermack-McKendrick set-up is formalized in the equations of Figure 1A.

As a prelude to the analytic discussion, let us trace through some simple state transitions. For instance, individuals susceptible to the bug and fear, *S*, flow into the Infected (with pathogen only) pool *I_P_* at rate 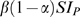 and into the pool infected with fear only *I_F_* at rate 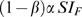. Similarly, those who self-isolated out of fear only, denoted *R_F_* (removed through Fear) return to the *S* pool at rate *HR_F_*, where *a constant H* would yield exponential decay of individual fear, *ceteris paribus. A time-dependant H is considered below*. This most elementary form of the model assumes constant population as all the right-hand sides sum to zero, and clearly subsumes the classic SI and SIR models. In parallel with this mathematical model, we also built an equivalent ABM, to be used as the basis for the with-flight extensions discussed below. All the following points pertaining to the differential equations also apply to the parallel ABM.

### Fear and Disease Uncoupled

Setting 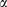 (fear) equal to zero (and barring removals) yields the standard S-curves (i.e., a declining susceptible S-curve and a rising disease infection S-curve. In turn, if we reverse things, setting disease transmission (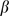) to zero, and fear transmission (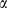) to a positive value, we generate a pure fear epidemic with no underlying disease. The Salem witch hunt would be one example, though there are innumerable further ones.

### Fear and Disease Coupled

**Figure 2:**
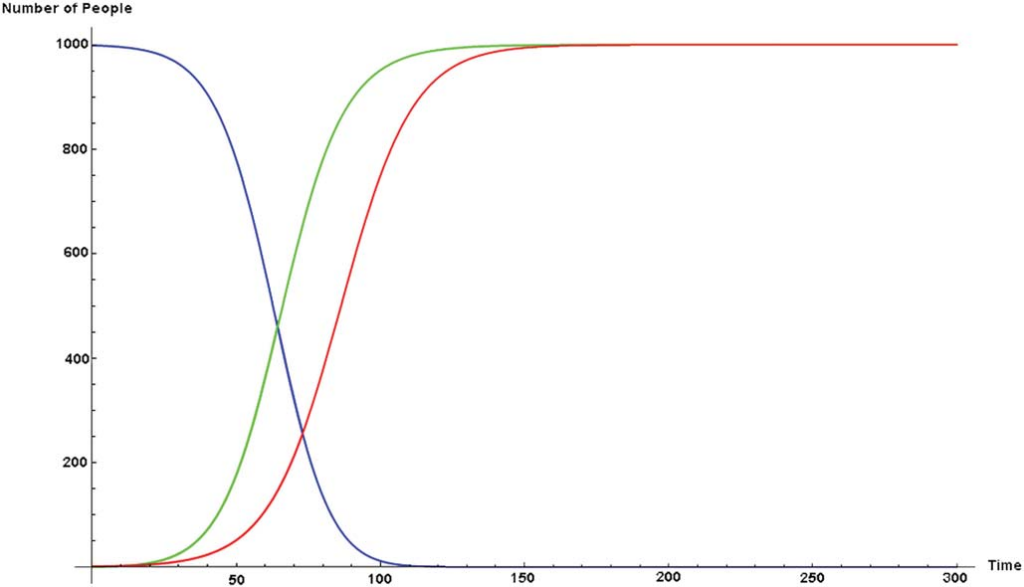
Here we provide the coupled case with α = β = 0.0008. One would expect that if we set *α* = *β*, the disease and fear epidemic S-curves should coincide, but this is not the case. Fear (the green curve) precedes disease (the red curve).

These results seem reasonably predictable, and are symmetrical to one another: at 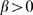 and 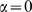, we get S-curves of disease. Reverse these settings 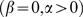 and we get the strictly analogous pair of S-curves for fear. Surely one would expect that if we set 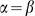, the disease and fear epidemic S-curves should coincide. Is this what happens? Not always, as shown in Figure 2, with 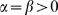.

*Ceteris paribus*, the fear epidemic is faster then the bug epidemic. Why? The reason is that there are more pathways by which to contract fear than there are to contract bug. One can contract disease from contact with either of *two* pools: I*_P_* or I*_PF_*. But one can contract Fear by contact with any one of *three* pools: I*_B_*, I*_BF_*, or I*_F_*. Obviously, once there is any fear, the latter three is a bigger set with which contact is more likely. This is a numerical example showing the possibility of an important asymmetry. But it is simply an existence result. What is the general relationship?

### Reproductive Rates of Fear and Disease

One measure of epidemic speed is the basic reproductive number: R_0_. This is defined as the expected number of secondary cases from a typical infectious individual during the entire period of their infectiousness in a completely susceptible population. The basic reproductive number of either the pathogen or fear can be found by calculating the spectral radius of the next generation operator. Diekmann et al. describes this procedure for estimating R_0_ about the disease-free equilibrium [7]. The basic reproductive number of the pathogen as a function of the transmission coefficient and rates of recovery or withdrawal from contact from the above system of equations is:
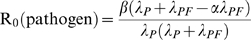
(1)


Two types of individuals are infectious with the pathogen, I*_P_* and I*_PF_*. The average residence time in each of these states is 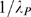 and 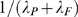, respectively. Individuals in these states will infect others at a rate of 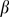 per unit time. R_0_(pathogen) can be interpreted as a weighted sum of the product of 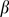 and the residence times in the two infectious states weighted by the fraction of those that become infected by the pathogen who transit to 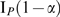 and 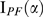.

The basic reproductive number of fear is given by:
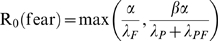
(2)


The first term above, 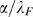, is the product of the transmission coefficient of fear, 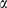, and the duration of the infectious period of fear, 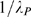. This is the classical form of the basic reproductive number for a pathogen in an SIR model with a closed population. The second term of equation 2 is greater than the first term only when the ratio of the infectious period of fear (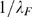) to the infectious period of those with pathogen and fear, 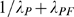, is less than the transmission coefficient of the pathogen, 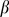. In this case, the basic reproductive number of the pathogen exceeds the basic reproductive number of fear.

In the case where 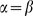 and 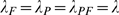 we find that fear spreads faster than disease, as 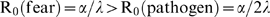 (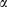 and 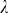 are both non-negative). When all three rate constants and the transmission coefficients differ from one another, the basic reproductive number of fear exceeds the basic reproductive number of the pathogen precisely when:
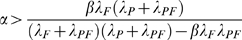
(3)


In the absence of fear or pathogen, these models collapse to SIR or SIRS models in pathogen or fear. So, in the absence of transmissible fear, 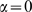, the R_0_(pathogen) equation 1 reduces to the classical R_0_ of 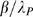. In the absence of pathogen, the model collapses to an SIRS model of fear due to the recovery of individuals to the susceptible state and R_0_(fear) is 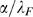. In spatially extended settings where fear may inspire long-range migration, the possibility of fear propagating faster than bug will prove highly consequential.

### Plausible Behavioral Mechanism for Multiple Waves of Infection in 1918

**Figure 3:**
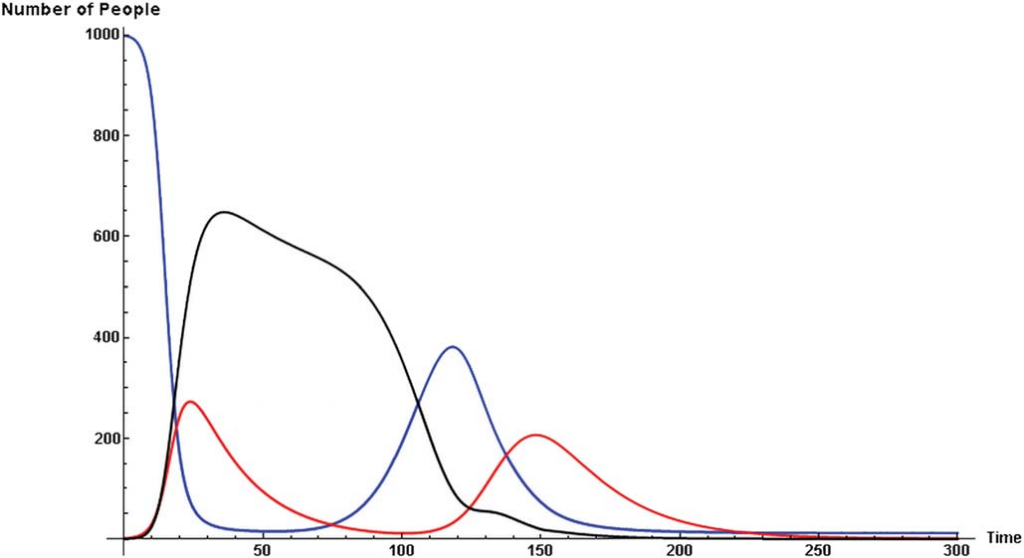
In the idealized run of figure 3, susceptible individuals (blue-curve) self-isolate (black curve) through fear as the infection of disease proper grows (red curve). Emboldened by the falling disease incidence, these susceptibles return (prematurely) to circulation (the blue hump). But, this offers fuel to the remaining embers of infection (at time 100), and a second wave ensues.

Finally, multiple waves of infection of the sort that occurred in 1918 are easily generated in this model. For example, it suffices to 
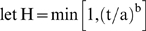
, with a = 150 and b = 8. In the idealized run of figure 3, susceptible individuals (blue-curve) self-isolate (yellow curve) through fear as the infection of disease proper grows (red curve). Emboldened by the falling disease incidence, these susceptibles return (prematurely) to circulation (the blue hump). But, this offers fuel to the remaining embers of infection (at time 100), and a second wave ensues. This reflects the counterintuitive and crucial insight of the original Kermack-McKendrick model, that the epidemic threshold is *not* the infective level, but rather the susceptible one. Authorities in 1918 did not have the benefit of this insight, and in a number of cities lifted quarantines prematurely, with the same effect: multiple waves.

### Part II: Spatial Propagation in the Agent-Based Computational Model with Flight

As noted earlier, most research in epidemiology does not take into consideration the possibility of behavioral adaptations that are prevalence-dependent (see above). Those models that *do* include prevalence-dependent behavior almost exclusively assume behavioral changes that are *depressive* in their effect on the epidemic, protective self-isolation (sequestration) being the most common.

However, research on mass behavior during crises, and even epidemics specifically, records another common behavioral response—*flight*. Historical cases of flight from epidemics are numerous, dating back at least as far as Medieval Europe, where the morality of fleeing from the Plague was a central and divisive topic among early modern Jesuits [8]. In the 19^th^ century, large scale flight was a common behavioral response to urban epidemics of cholera and yellow fever. For example, more than 25,000 residents (almost half the population) fled Memphis when yellow fever struck in summer 1878, and as the fever spread through the South the highest incidence was in cities directly along railroad lines leading out of Memphis [9]. Within days of cholera's appearance in Cairo in 1831, the Nile “swarmed with craft of every description filled with refugees from the stricken city” as a mass exodus began [10]. Cholera arrived in North America for the first time in 1832, carried by Irish immigrants fleeing the epidemic in Ireland. As it spread rapidly through the Midwest and Northeast of the United States, flight was common: “the appearance of cholera in even the smallest hamlet was the signal for… headlong flight, spreading the disease throughout the surrounding countryside” [11]. Flight was also a response to 20^th^ century epidemics such as polio, influenza, and plague. In some cases, fear alone was sufficient to cause flight (even in the absence of any confirmed disease) and “sociogenic” illness—for example in Surat India in 2006, and Melbourne Australia in 2005 [12].

The potential for flight as a behavioral response to disease prevalence has important consequences for epidemic modeling. Unlike protective sequestration, flight has the potential to *increase* mixing in the short term, and across spatial regions (even if it ultimately removes individuals from circulation *locally*). In the model developed below, we expand the behavioral response repertoire of agents infected with fear to include both flight and protective self-isolation. For now, a specific behavioral response is a characteristic of each individual—some agents always flee when afraid, others hide. We explore the impact of differing levels of flight on the epidemic dynamics.

Of course, one could in principle formulate this as a high dimensional meta-population ODE model with many patches and coupling coefficients. (Formulation as a reaction diffusion system on a spatial continuum might also be possible.) However, with the inclusion of both self-isolation and flight, the result would be a fairly opaque ODE system. As noted earlier, we built an ABM that mimics the 7D differential equation system discussed above. Now we extend it to include space and flight, in addition to all the features of the earlier ODE model. The extended agent-based version will prove very transparent, and quickly yields the main result: *even a small level of flight can dramatically affect contagion dynamics*.

### Set-up

The agent model with self-isolation and flight takes place on a 2D lattice (i.e., a large checkerboard) where time is discretized into many brief periods. During each time period agents typically move a short distance in a random walk and then contact a random local agent (if any are near). That contact spreads both the bug and the fear according to 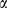 and 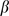. However, an agent who has contracted fear may or may not adapt its movement and contact behaviors.

The model has three types of agents, representing three different characteristic responses to fear. The first type, “fleers,” respond to fear by selecting a new location some distance from their current position on the lattice and moving there as fast as possible. For the runs reported here, this is the site 15 sites south of the agent's current location. We are measuring corner-to-corner spread. This distance is 120√2, so 15 sites is roughly 9% of the diagonal distance. This is far too small for any single fleer to be driving the main spread results, and should allay any such concern. Upon reaching the goal location this agent will recover from fear and his movement rule reverts to a random walk (with radius of one site). The second type, “hiders,” respond to fear by removing themselves from circulation for a specified number of iterations (during which they neither move nor contact other agents). The third type, “ignorers,” never change their movement or contact behavior.

Parameters of the model include the size of the lattice, the total population of agents, the distribution of agent types, movement and contact radii, the transmission rates of fear and bug, the duration of sickness, the distance fled, and the duration sequestered. The parameters used for all of the simulation experiments discussed in this section are: 1800 agents on a 120×120 lattice, alpha = 0.11, beta = 0.1, lambda = 0.015, illness duration = 100 periods, and fear duration = 800 periods. A model run ends when no more agents are infected.

## Results and Discussion

The results from this agent model highlight the importance of flight as a topic for research—even a small amount of flight can have a dramatic impact on epidemic dynamics.

**Figure 4:**
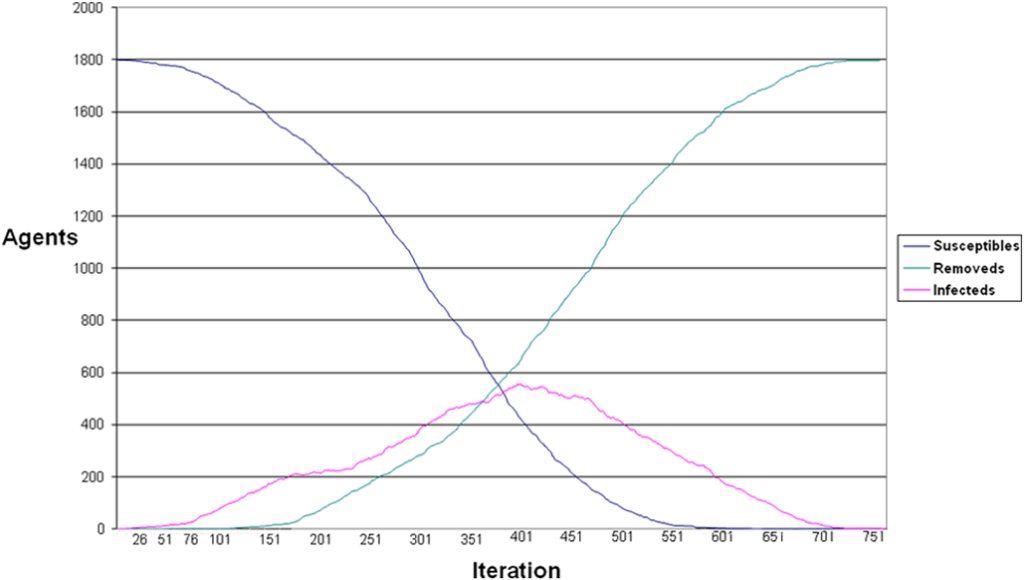
We consider a simple form of the model in which fear propagates, but no one adapts their behavior in response. All agents are “ignorers.” Parameterized in this way, the model produces quintessential SIR curves.

First, to establish a baseline, we consider a simple form of the model in which fear propagates, but no one adapts their behavior in response. All agents are “ignorers”. Parameterized in this way, the model produces quintessential SIR curves, such as those shown in figure 4.

**Figure 5:**
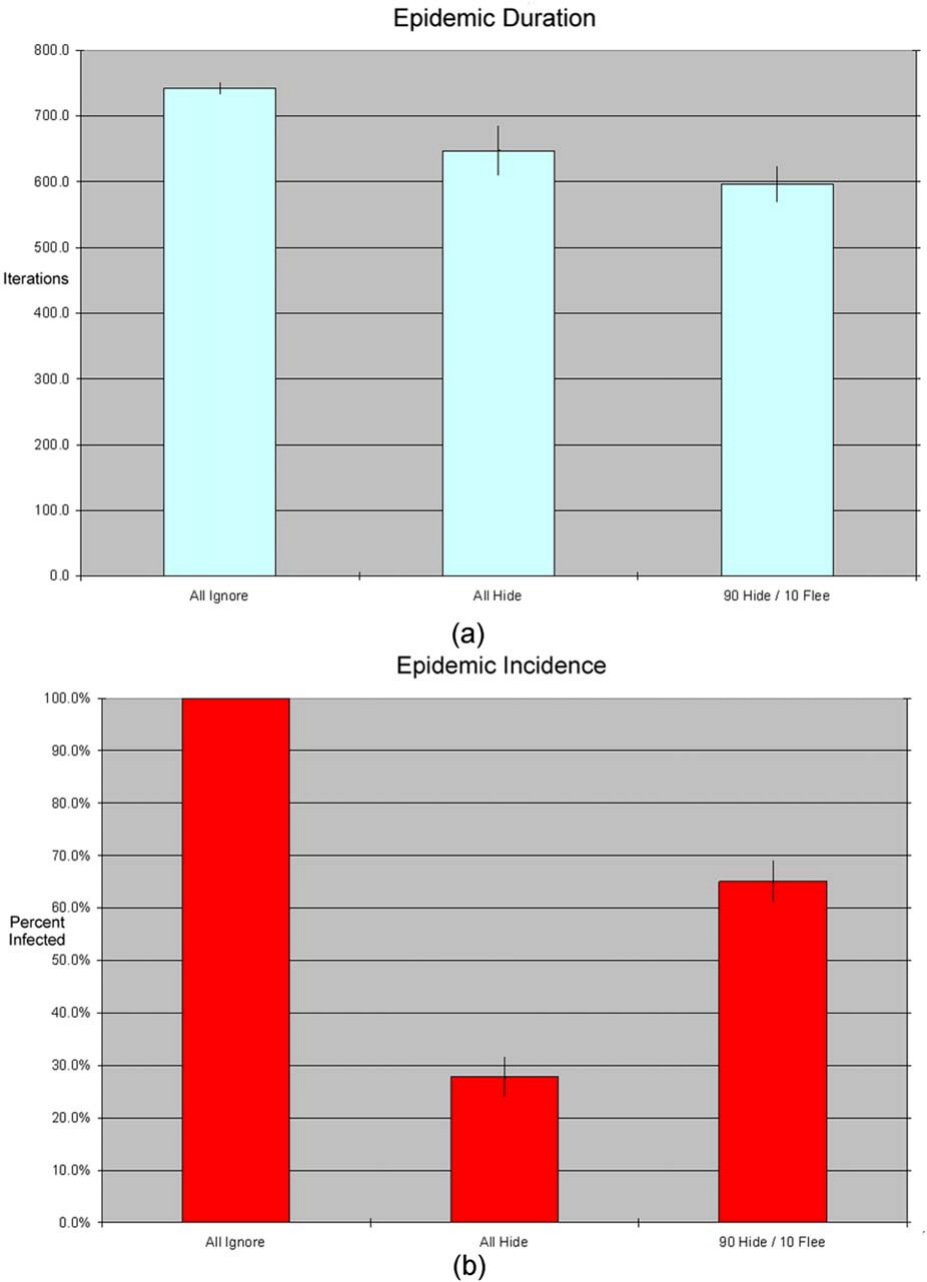
(A&B): Epidemic duration and total incidence under three different parameter settings. Each bar in the chart represents an average across 30 simulation runs for a given parameter setting, with standard error range. When all agents hide, the epidemic is shorter and has substantially lower incidence that with no adaptive behavior. When a small percentage of agents flees (with the majority hiding), however, incidence goes up substantially even as the duration falls farther.

In a 30-run analysis, the average epidemic duration with all agents set to “ignorers” is 742.1 periods (SE 9.5), and average total incidence is 99.9%. (See the leftmost bars of Figures 5a and b, respectively)

In the next version of the model we replace the population of “ignorers” with a population of “hiders.” Replacing the population with “hiders” drastically reduces incidence to an average of 27.8% and stops the epidemic earlier (in an average of 647 rounds). A population who systematically hides from an incoming epidemic will suffer many fewer cases of disease. See the middle bars of Figure 5a and b below.

Next, we introduce flight, but only a small amount—90% of agents still respond to fear by hiding (removing themselves from circulation); the remaining 10% flee. How does this small proportion of flight affect incidence and duration of the epidemic?

As Figure 5 shows, even this small amount of flight dramatically *increases* the size and speed of the epidemic (comparing the rightmost bars of 5a and b to the middle ones). Average incidence in the population is 64% and the average epidemic duration is 595 time periods. This small proportion of fleeing agents causes the population to suffer more than 2.3 times as many disease cases as in the all-hiders configuration.

**Figure 6:**
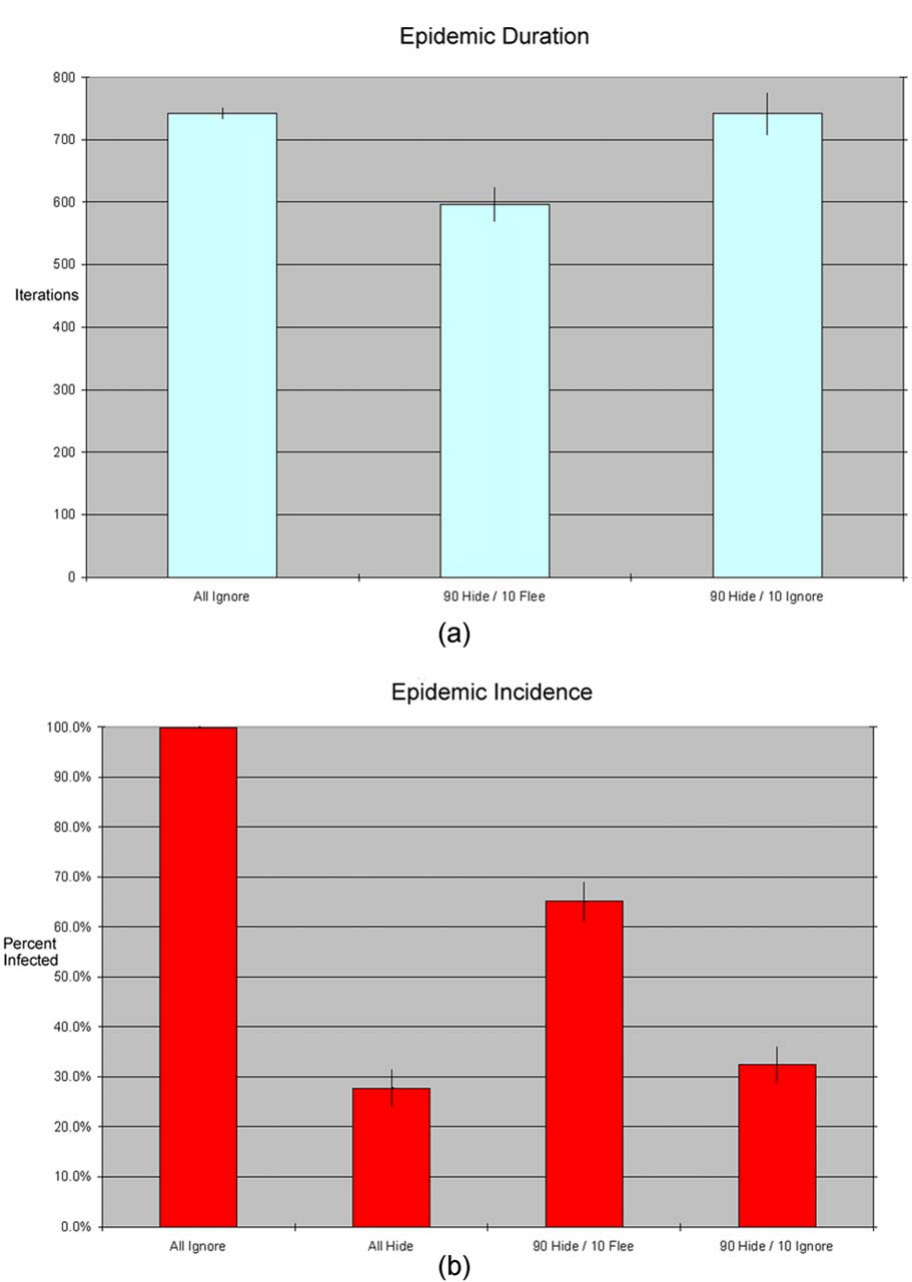
(A&B): A comparison of epidemic duration and total incidence with 10% “fleers” versus 10% “ignorers.” As before, each bar in the chart represents an average across 30 simulation runs for a given parameter setting, with standard error range. The runs with 10% “ignorers” have similar incidence to runs with 100% “hiders,” and similar duration to runs with 100% “ignorers.” By contrast, the runs with 10% “fleers” have much higher incidence and lower duration.

Of course, the 10% of agents who are fleeing are also not hiding. By remaining in circulation, even ignoring (neither fleeing nor hiding) agents should have an inflammatory effect on the epidemic. So, is it the flight or simply the increased circulation from non-hiding which is driving the previous result? To answer this question, we ran the simulation with 90% “hiders” and 10% “ignorers.” As Figure 6 illustrates, the results from these runs differ noticeably from the runs with actual flight: we observed an average incidence of 32% and an average epidemic duration of 640 time periods. Flight has a substantial impact above and beyond increasing the number of “non-hiders”.

**Figure 7:**
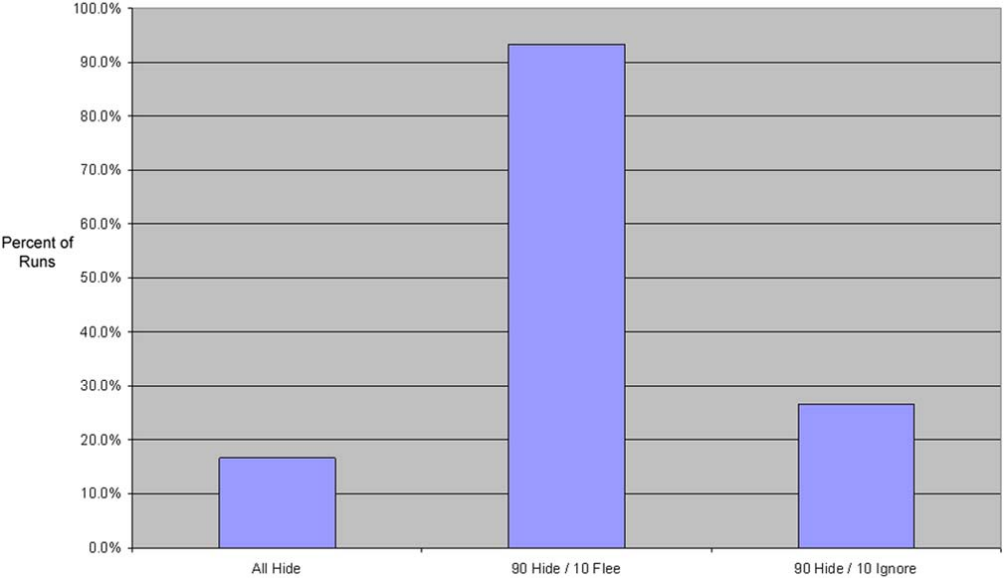
The percentage of runs (out of 30) for each parameter setting in which the epidemic spreads fully across the landscape, from an index case in one corner of the lattice all the way to the opposite corner.

Not only does flight increase incidence dramatically, but it also increases the rapidity and geographic scope of the epidemic. One way to measure the geographic spread of the bug is to begin the epidemic with an index case in one corner of the 2D lattice, and observe if and when the bug reaches the far diagonal corner. Figure 7 shows that epidemics rarely spread fully across the lattice with no flight—but almost always spread fully across the lattice with even a small amount of flight. Specifically, when merely 10% flee, the epidemic reaches the far corner an average of 92% of the time. However, when no agents flee, the epidemic only reaches the far corner 17% (all hiders) or 27% (90% hide / 10% ignore) of the time.

**Figure 8:**
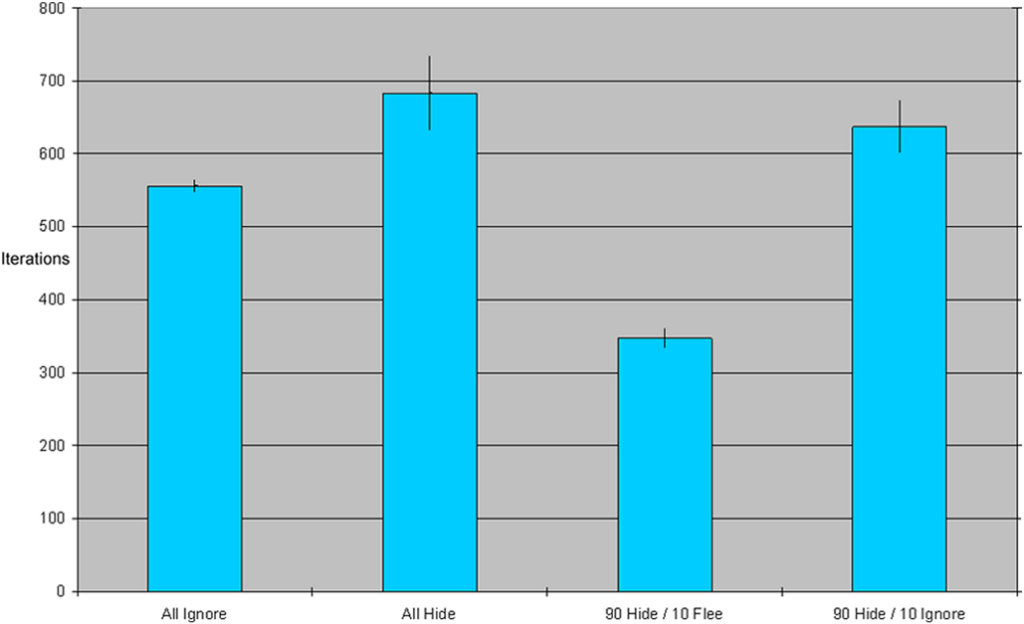
In rare cases where the epidemic spreads fully across the lattice without flight, it takes much longer to do so than in cases with flight. Without flight the epidemic takes roughly 600 time periods to cross the lattice.

Furthermore, in rare cases where the epidemic spreads fully across the lattice without flight, it takes much longer to do so than in cases with flight, as shown in Figure 8. Without flight the epidemic takes roughly 600 time periods to cross the lattice. When fight is allowed, the average run takes 340 time periods to traverse the lattice, almost twice as fast.

**Figure 9:**
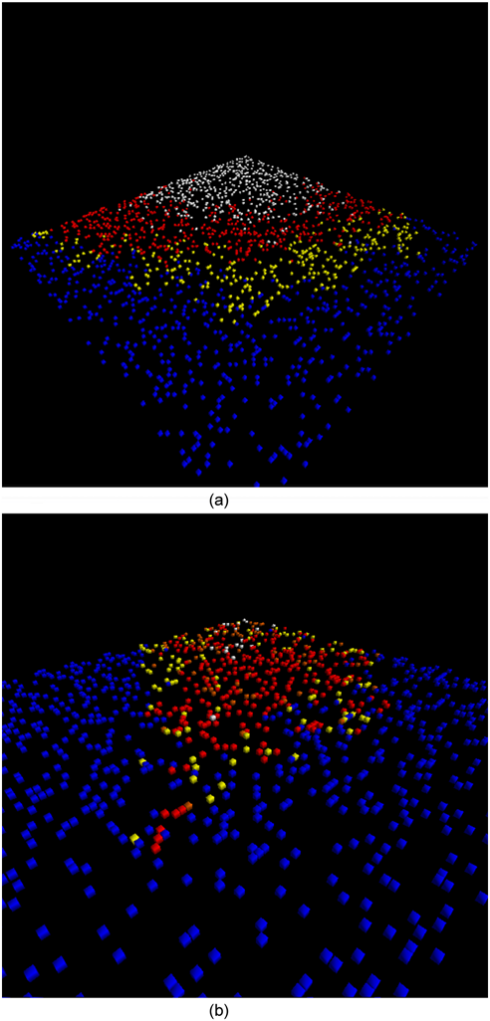
(A&B): Screenshots from the agent-based simulation model without and with flight. Each agent is represented by a colored dot on the lattice.

For an illustration of how flight spreads the epidemic quickly across the lattice, increasing both incidence and speed, see Figure 9. Blue dots represent susceptibles (infected with neither fear nor pathogen); yellow dots, infected with fear alone; orange dots, acting on fear; red dots, infected with pathogen; white dots, recovered.

In the first screen shot (9a), with no flight, yellow agents (infected with fear) form a moving buffer zone between the epidemic of pathogen and the susceptible agents. The latter thus have an opportunity to remove themselves from circulation because they are likely to contract fear *before* they are exposed to the pathogen (as per our earlier discussion of the fear R0), The second screenshot shows how a small amount of flight enables a few infected fleeing agents to pierce this buffer zone, introducing the pathogen quickly into the susceptible pool.

These specific quantitative results are summarized in Table 2. They are, of course, dependent on the specific parameters used above. But the larger qualitative point is robust. Behavioral adaptation need not damp the force of an epidemic. If flight is admitted, this form of “social distancing” can increase both the speed and size of an epidemic.

**Table 2:** Summary of flight results.

	Ignorers	Hiders	Fleeing agents	Mean time	Mean total incidence
**Baseline**	100%	0%	0%	742	99.9%
**Hiders**	0%	100%	0%	647	27.8%
**Flight**	0%	90%	10%	595	64.0%
**Fewer Hiders**	10%	90%	0%	640	35.0%

This exposition invites a great deal of further work, including development of the multi-patch (meta-population) ODEs with flight, full sensitivity analysis of the agent-based model, further “dialogue” between the two approaches, and calibration to historical cases.

However, the present effort clearly enforces the overarching point that *infectious disease models must incorporate behavioral adaptation*. In the development above, the adaptive repertoire is quite narrow, including only self-isolation and flight. But it can obviously be broadened substantially. Moreover, the model—while explored for contagious disease here—can be applied to a wide range of cases where momentous contagions of fear eventuate from events that are not themselves contagious, such as toxic chemical plumes or floods, fires, and earthquakes.
